# Machine learning for personalized antimicrobial susceptibility breakpoints (*Adaptive clinical breakpoint interpretation*)

**DOI:** 10.1093/jac/dkaf419

**Published:** 2025-11-12

**Authors:** Yinzheng Zhong, William Hope, Iain Buchan, Anoop Velluva, Alessandro Gerada, Conor Rosato, Peter L Green, Alex Howard

**Affiliations:** Department of Clinical Pharmacology & Therapeutics, University of Liverpool, Liverpool, UK; Department of Clinical Pharmacology & Therapeutics, University of Liverpool, Liverpool, UK; Department of Public Health, Policy & Systems, University of Liverpool, Liverpool, UK; Department of Clinical Pharmacology & Therapeutics, University of Liverpool, Liverpool, UK; Department of Clinical Pharmacology & Therapeutics, University of Liverpool, Liverpool, UK; Department of Clinical Pharmacology & Therapeutics, University of Liverpool, Liverpool, UK; Department of Mechanical and Aerospace Engineering, School of Engineering, University of Liverpool, Liverpool, UK; Department of Clinical Pharmacology & Therapeutics, University of Liverpool, Liverpool, UK

## Abstract

**Objectives:**

Infection diagnoses are critical to the personalized interpretation of EUCAST aminopenicillin breakpoints for Enterobacterales, but microbiology laboratories cannot predict diagnosis when specimens are received. Here, we assess whether machine learning could facilitate personalized antimicrobial susceptibility breakpoint reporting by predicting urinary tract infection (UTI) diagnoses.

**Methods:**

XGBoost models were trained using open-source electronic healthcare record data to predict complicated UTI in patients with Enterobacterales bacteriuria and to predict UTI in patients with Enterobacterales bacteraemia. These models were validated and used to provide simulated aminopenicillin dosing/regimen recommendations based on antimicrobial susceptibility results for patients with bacteriuria and bacteraemia in a holdout dataset. The main outcomes were the proportions of patients recommended appropriate aminopenicillin dosages/regimens according to EUCAST guidelines based on their diagnosis.

**Results:**

The area under the receiver operating characteristic curve was 0.62 for predicting both complicated UTI in patients with bacteriuria and UTI in patients with bacteraemia. In the simulation study, 79.3% (*n* = 276) and 72.7% (*n* = 8) of patients with ampicillin-susceptible Enterobacterales bacteriuria and bacteraemia, respectively, were recommended appropriate aminopenicillin dosages/regimens for their infection diagnosis according to EUCAST guidelines. Adjusting the probability threshold for predicting complicated UTI increased the proportion of appropriate recommendations in bacteriuria to 96.6% (*n* = 336).

**Conclusions:**

Using machine learning models to predict the probability of complicated UTI in patients with bacteriuria and the probability of UTI in patients with bacteraemia resulted in appropriate aminopenicillin dosages/regimens being recommended in most cases. These results provide proof-of-concept for how machine learning could facilitate the personalized implementation of EUCAST aminopenicillin breakpoints.

## Introduction

Antimicrobial susceptibility testing is fundamental for the delivery of safe and effective antimicrobial therapy.^1^ The MIC is a standardized measure of antimicrobial potency. A breakpoint is an MIC value that separates a population into groups with a high and low probability of a favourable clinical outcome.^2^ Breakpoint values are ordinarily population level estimates and therefore are counter to notions of individualized care. Ideally, breakpoints should be specific for an individual patient’s antimicrobial regimen, disease type [e.g. pneumonia versus urinary tract infection (UTI)], host characteristics (e.g. immunocompetent versus immunocompromised), density of infection, and other pathogen-related factors. To facilitate individualized care, new approaches are required to interpret breakpoints in a way that allows individual patients to maximally benefit from antimicrobial therapy.^3^

The revised EUCAST breakpoints for oral aminopenicillins (i.e. amoxicillin, ampicillin, and amoxicillin–clavulanic acid) state that MICs and disc susceptibility testing (DST) zone sizes for Enterobacterales should be interpreted based on whether a patient has an uncomplicated UTI (uUTI), complicated UTI (cUTI; i.e. a UTI that extends beyond the bladder or for which there are complicating factors such as pregnancy, immunosuppression, or male sex), or an infection from a non-urinary source (e.g. biliary tract infection).^4^ Based on this interpretation, patients with uUTI, cUTI, and non-urinary foci of infection should receive standard-dose oral amoxicillin, high-dose oral amoxicillin, and high-dose oral amoxicillin as part of combination antibiotic therapy, respectively. However, implementing these recommendations is challenging for diagnostic microbiology laboratories because they lack ready access to the clinical information required to determine the likely disease and apply the most appropriate breakpoint.

The increased use of electronic health records (EHRs) could afford new opportunities to use patient data to support clinical decisions globally. Recent advances in machine learning methodology and computing power have also increased interest in potential applications in clinical microbiology for image analysis, molecular diagnostics, and process automation.^5–8^ Machine learning could also enable diagnostic microbiology laboratories to apply breakpoints on an individualized basis. Here, we predicted UTI in patients with bacteraemia and cUTI in patients with bacteriuria using real-world EHR data and machine learning methods. Our goal was to provide proof-of-concept for an approach to support the appropriate implementation of EUCAST breakpoints for aminopenicillins in diagnostic microbiology laboratories and to serve as a pathfinder for similar applications for other antibiotics.

## Methods

### Dataset

The Medical Information Mart for Intensive Care-IV (MIMIC-IV) version 2.2 was used as the dataset. MIMIC-IV is an open-source EHR dataset from Beth Israel Deaconess Medical Center in Boston, MA (https://physionet.org/content/mimiciv/2.2/).^9–11^ The dataset contains inpatient and outpatient data for patients admitted to intensive care or the emergency department 2008–2019. The study complied with required ethical regulations (the PhysioNet Credentialed Health Data Use Agreement 1.5.0 and Health Data License). The dataset was cleaned using RStudio Version 2023.12.1 + 402 (2023.12.1 + 402)—antimicrobial susceptibility results were transposed from rows to columns using the MIMER package (https://cran.r-project.org/web/packages/MIMER/index.html), and EUCAST intrinsic resistance patterns were imputed using EUCAST rules encoded into the ‘AMR’ package (https://cran.r-project.org/web/packages/AMR/index.html). EUCAST expected susceptible phenotypes were simulated using a Bayesian approach with evidence-based priors described previously.^12^

### Model development

Two binary prediction Extreme Gradient Boosting (XGBoost) models were developed. XGBoost is a machine learning algorithm that uses additive decision trees to build predictive discriminative models.^13^ The first model (hereafter referred to as the ‘urine culture model’) was used to predict whether patients with bacteriuria had uUTI or cUTI; the second (hereafter referred to as the ‘blood culture model’) predicted whether patients with bacteraemia had a urinary or non-urinary focus of infection. These outcomes were determined using diagnostic coding data from the hospital admission.

For the urine culture model, cUTI was defined as a UTI coded for that admission alongside a coded urinary obstruction, structural/functional abnormality, presence of a urinary catheter/instrumentation, and/or the same organism grown in urine and blood during the same admission.^14^ Patients who did not have a coded diagnosis of UTI for that admission and/or did not grow organisms in their urine were excluded from the study. Patients who were pregnant, male, or on immunosuppressive drugs were also excluded because these cohorts have cUTI by definition.

For the blood culture model, a urinary source of infection was defined as patients with bacteraemia who had a UTI of any kind coded for that admission. Predictor variables/features eligible for inclusion in both models were any that occurred up to and including triage for that hospital admission. Exact features used in this study can be found on our GitHub repository (https://github.com/AMRx-LIV/ADAPT-DSR).

### Data preprocessing

For the urine culture and blood culture datasets in turn, data were divided into two subsets. A training dataset comprised 80% of the total dataset and was used to train and select a model based on the performance of candidate models in a 10-fold cross-validation (i.e. each fold was trained on 72% of the total data and tested on 8%). The remaining 20% of the total data (hereafter referred to as the ‘holdout dataset’) was used to validate the selected model’s predictive performance and to perform a microsimulation study (see below).

The min–max scaler, which is a normalization technique that preprocesses the data features into a range between zero and one, was applied to normalize numerical data on a column-wise basis. The formula for the min–max scaling is as follows:


$$x_{scaled}=\frac{x-x_{\min}}{x_{\max}-x_{\min}}.$$

Given that the holdout data were treated as unseen to simulate conditions for real-world application of the approach, minimum and maximum values observed from the training dataset were used to scale the holdout dataset (scaled values outsize the zero to one range were therefore theoretically possible in the holdout dataset). Categorical data were one-hot encoded by creating a separate variable with a zero (absence) or one (presence) value for each variable class. Other than the intrinsic resistance and expected susceptible phenotype patterns mentioned above, missing predictor variable data were not imputed—an in-built XGBoost adaptive handling mechanism was instead employed where loss minimization during training is used to determine the optimal default decision tree branch to follow when missing data elements are encountered during final model training.^15^

### Training and testing

The XGBoost models were configured to have tree depth equal to 3, a sub-sample set to 0.7, and a learning rate set to 0.005. For the urine culture and blood culture datasets in turn, 10-fold cross-validation was performed on the training dataset to assess how models performed across different train–test splits in the dataset. The trained model instance with the best area under receiver operating characteristic (AUROC) in the 10-fold cross-validation was selected for final performance validation against the holdout dataset. Consequently, two models were validated—one for the urine culture data and one for the blood culture data. To address sample imbalance, sample weights were trialled but did not show significant impact on predictive performance in cross-validation (the AUROC was unchanged to two decimal places in both models). Sample weighting was therefore not used for the final analysis. Metrics used to measure performance were AUROC, average precision (AP), F1 score, correlation (Corr), precision (P), recall (R), and accuracy (Acc). ROC and precision-recall curves were also plotted for both the cross-validation and final holdout validation.

### Fairness analysis and feature importance

Machine learning models trained on EHR data may inherit biases from data collection methodologies or underlying population disparities. A fairness analysis was used to compare each model’s performance in different demographic groups.^16^ Race, sex, and age groups were used for the fairness test. Small racial sub-groups were merged into larger groups to avoid an insufficient number of samples, and ages were grouped into 10 year ranges. Shapley additive explanation (SHAP) values from the training of the best model in cross-validation were used to show feature contributions across all data points in the prediction process, providing a quantitative measure of how much each feature influenced each model’s predictions.^17,18^

### Microsimulation study and outcomes

**Figure 1.**
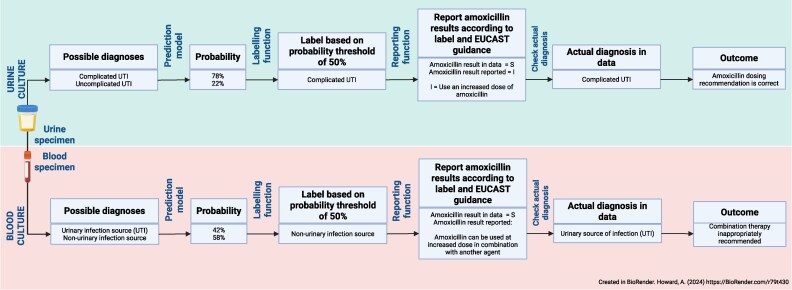
Design of the microsimulation study.

The projected effects of antimicrobial prescribing were assessed using a microsimulation approach on the holdout dataset, as illustrated in Figure 1, where the breakpoint interpretation decision was made according to EUCAST guidelines on aminopenicillin reporting and the disease predictions of the urine/blood culture models.^19^ The outcomes assessed were the proportions of patients with Enterobacterales bacteriuria and bacteraemia who were recommended the correct amoxicillin treatment regimen based on the breakpoint interpretation decision. These outcomes were assessed at a default probability decision threshold of 0.5 (i.e. a diagnosis was predicted if its predicted probability was ≥0.5). A sensitivity analysis was then performed where the proportions of correct, excessive, and insufficient amoxicillin regimens were measured across a range of 11 probability thresholds between 0 and 1 inclusive—these results were then plotted for visualization purposes. To ascertain the potential impact of the approach on aminopenicillin use in the population studied, a sub-analysis was performed where the same outcomes were assessed only in patients who were on amoxicillin or amoxicillin–clavulanic acid at the time of the urine specimen or in the 7 days afterwards.

To demonstrate the potential applicability of the approach to other antimicrobial agents, a sensitivity microsimulation analysis was performed to assess the potential impact of model predictions on the interpretation of nitrofurantoin susceptibility in the urine culture dataset across all urinary organisms (nitrofurantoin breakpoints are only reportable for uncomplicated UTI in EUCAST guidelines because of the drug’s relatively poor tissue penetration).^4,20^ Where the model predicted ≤0.5 probability of complicated UTI, susceptible (‘S’) nitrofurantoin results were reported as susceptible. Where the model predicted >0.5 probability of cUTI, results were reported as resistant (‘R’). The proportion of patients for which this interpretation was correct was measured in the same way as the main microsimulation study.

## Results

### Model performance

A total of 9479 patients were eligible for study in the urine culture data and 1947 in the blood culture data. The characteristics of the study population are outlined in Table 1.

**Table 1.** Summary statistics for the datasets. Based on number of samples instead of subjects

Measure(s)		Urine culture dataset	Blood culture dataset
Age, years, median (IQR)		69 (56–80)	64 (52–74)
Gender female (*F*), *n* (%)/total		12 008 (100.00%)/12 008	1237 (53.34%)/2319
Race, *n* (%)			
White		8121 (67.63%)	1510 (65.11%)
Black/African American		1433 (11.93%)	304 (13.11%)
Unknown		556 (4.63%)	58 (2.50%)
Other		329 (2.74%)	64 (2.76%)
Hispanic/Latino—Puerto Rican		154 (1.28%)	46 (1.98%)
White—Other European		154 (1.28%)	30 (1.29%)
Hispanic or Latino		141 (1.17%)	26 (1.12%)
White—Russian		136 (1.13%)	24 (1.03%)
Asian—Chinese		123 (1.02%)	21 (0.91%)
Asian		109 (0.91%)	33 (1.42%)
Unable to Obtain		99 (0.82%)	17 (0.73%)
Hispanic/Latino—Dominican		94 (0.78%)	26 (1.12%)
*Additional categories omitted for brevity*			
Organism grown, *n* (%)			
(Unknown genus)		3944 (32.84%)	31 (1.34%)
*Escherichia*		3370 (28.06%)	318 (13.71%)
*Enterococcus*		1448 (12.06%)	259 (11.17%)
*Klebsiella*		993 (8.27%)	158 (6.81%)
*Staphylococcus*		538 (4.48%)	977 (42.13%)
*Proteus*		403 (3.36%)	16 (0.69%)
*Pseudomonas*		340 (2.83%)	63 (2.72%)
*Streptococcus*		216 (1.80%)	101 (4.36%)
*Enterobacter*		144 (1.20%)	47 (2.03%)
*Citrobacter*		125 (1.04%)	12 (0.52%)
*Gardnerella*		105 (0.87%)	—
*Corynebacterium*		94 (0.78%)	45 (1.94%)
*Candida*		51 (0.42%)	116 (5.00%)
Other		235 (1.96%)	175 (7.55%)
Antibiotic, *n* (%)	AST Result		
Ampicillin	I	56 (0.47%)	1 (0.04%)
	R	6657 (55.44%)	1032 (44.5%)
	S	2729 (22.73%)	236 (10.18%)
	NA	2566 (21.37%)	1050 (45.28%)
Ampicillin/sulbactam	I	617 (5.14%)	44 (1.9%)
	R	4832 (40.24%)	802 (34.58%)
	S	4041 (33.65%)	509 (21.95%)
	NA	2518 (20.97%)	964 (41.57%)
Amoxicillin/clavulanic acid	R	3915 (32.6%)	667 (28.76%)
	S	2879 (23.98%)	406 (17.51%)
	NA	5214 (43.42%)	1246 (53.73%)
Piperacillin/tazobactam	I	62 (0.52%)	25 (1.08%)
	R	3265 (27.19%)	552 (23.8%)
	S	5762 (47.98%)	767 (33.07%)
	NA	2919 (24.31%)	975 (42.04%)
Cefalexin	R	4905 (40.85%)	809 (34.89%)
	S	313 (2.61%)	246 (10.61%)
	NA	6790 (56.55%)	1264 (54.51%)
Cefazolin	I	49 (0.41%)	NA
	R	5831 (48.56%)	NA
	S	3667 (30.54%)	461 (19.88%)
	NA	2461 (20.49%)	908 (39.15%)
Cefuroxime	I	77 (0.64%)	2 (0.09%)
	R	4625 (38.52%)	752 (32.43%)
	S	892 (7.43%)	276 (11.9%)
	NA	6414 (53.41%)	1289 (55.58%)
Cefoxitin screening	R	4511 (37.57%)	721 (31.09%)
	S	313 (2.61%)	246 (10.61%)
	NA	7184 (59.83%)	1352 (58.3%)
Ceftriaxone	I	27 (0.22%)	3 (0.13%)
	R	5460 (45.47%)	911 (39.28%)
	S	4254 (35.43%)	496 (21.39%)
	NA	2267 (18.88%)	909 (39.2%)
Ceftazidime	I	63 (0.52%)	19 (0.82%)
	R	5581 (46.48%)	1639 (70.68%)
	S	4388 (36.54%)	307 (13.24%)
	NA	1976 (16.46%)	354 (15.27%)
Cefpodoxime	R	4160 (34.64%)	642 (27.68%)
	S	313 (2.61%)	246 (10.61%)
	NA	7535 (62.75%)	1431 (61.71%)
Cefepime	I	80 (0.67%)	16 (0.69%)
	R	4536 (37.77%)	743 (32.04%)
	S	5092 (42.41%)	626 (26.99%)
	NA	2300 (19.15%)	934 (40.28%)
Ertapenem	R	3105 (25.86%)	606 (26.13%)
	S	310 (2.58%)	224 (9.66%)
	NA	8593 (71.56%)	1489 (64.21%)
Meropenem	I	21 (0.17%)	6 (0.26%)
	R	2806 (23.37%)	557 (24.02%)
	S	5497 (45.78%)	702 (30.27%)
	NA	3684 (30.68%)	949 (40.92%)
Levofloxacin	I	1 (0.01%)	3 (0.13%)
	R	4067 (33.87%)	572 (24.67%)
	S	127 (1.06%)	198 (8.54%)
	NA	7813 (65.06%)	1546 (66.67%)
Ciprofloxacin	I	68 (0.57%)	10 (0.43%)
	R	4136 (34.44%)	677 (29.19%)
	S	3818 (31.8%)	482 (20.78%)
	NA	3986 (33.19%)	1150 (49.59%)
Gentamicin	I	81 (0.67%)	42 (1.81%)
	R	4762 (39.66%)	613 (26.43%)
	S	4950 (41.22%)	808 (34.84%)
	NA	2215 (18.45%)	856 (36.91%)
Amikacin	I	5 (0.04%)	NA
	R	4261 (35.48%)	NA
	S	381 (3.17%)	67 (2.89%)
	NA	7361 (61.3%)	1751 (75.51%)
Trimethoprim/sulfamethoxazole	R	4126 (34.36%)	NA
	S	3878 (32.3%)	505 (21.78%)
	NA	4004 (33.34%)	1198 (51.66%)
Nitrofurantoin	I	758 (6.31%)	NA
	R	3968 (33.04%)	NA
	S	4650 (38.72%)	NA
	NA	2632 (21.92%)	2094 (90.3%)
Colistin	R	6661 (55.47%)	1652 (71.24%)
	S	4930 (41.06%)	601 (25.92%)
	NA	417 (3.47%)	66 (2.85%)
Tigecycline	R	3411 (28.41%)	223 (9.62%)
	S	2236 (18.62%)	1308 (56.4%)
	NA	6361 (52.97%)	788 (33.98%)
Vancomycin	I	1 (0.01%)	1 (0.04%)
	R	8577 (71.43%)	913 (39.37%)
	S	1626 (13.54%)	1171 (50.5%)
	NA	1804 (15.02%)	234 (10.09%)

AST, antimicrobial susceptibility testing.

**Figure 2.**
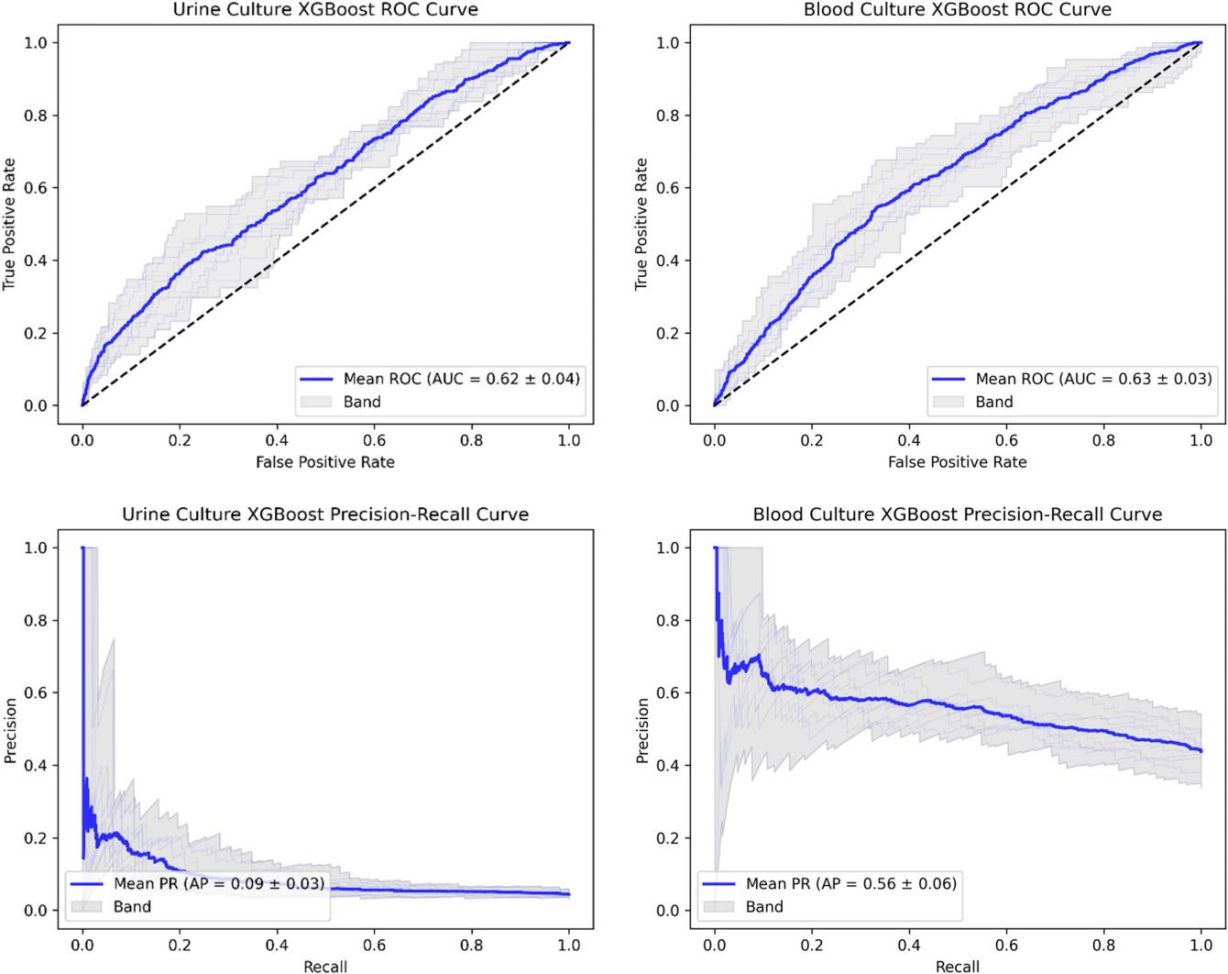
Receiver operating characteristic (ROC) curves (top) and precision-recall (PR) curves (bottom) from the 10-fold model training cross-validation performed in the urine culture dataset (left) and the blood culture dataset (right). The blue curves represent the mean values from the 10-fold predictions, with grey curves illustrating fold-specific curves and the band covering the upper and lower bounds. Mean values for area under the ROC curve (AUC) and average precision (AP) are included in inset boxes with their standard deviations. Dotted lines in ROC plots represent an AUC of 0.5 (random predictions).

**Figure 3.**
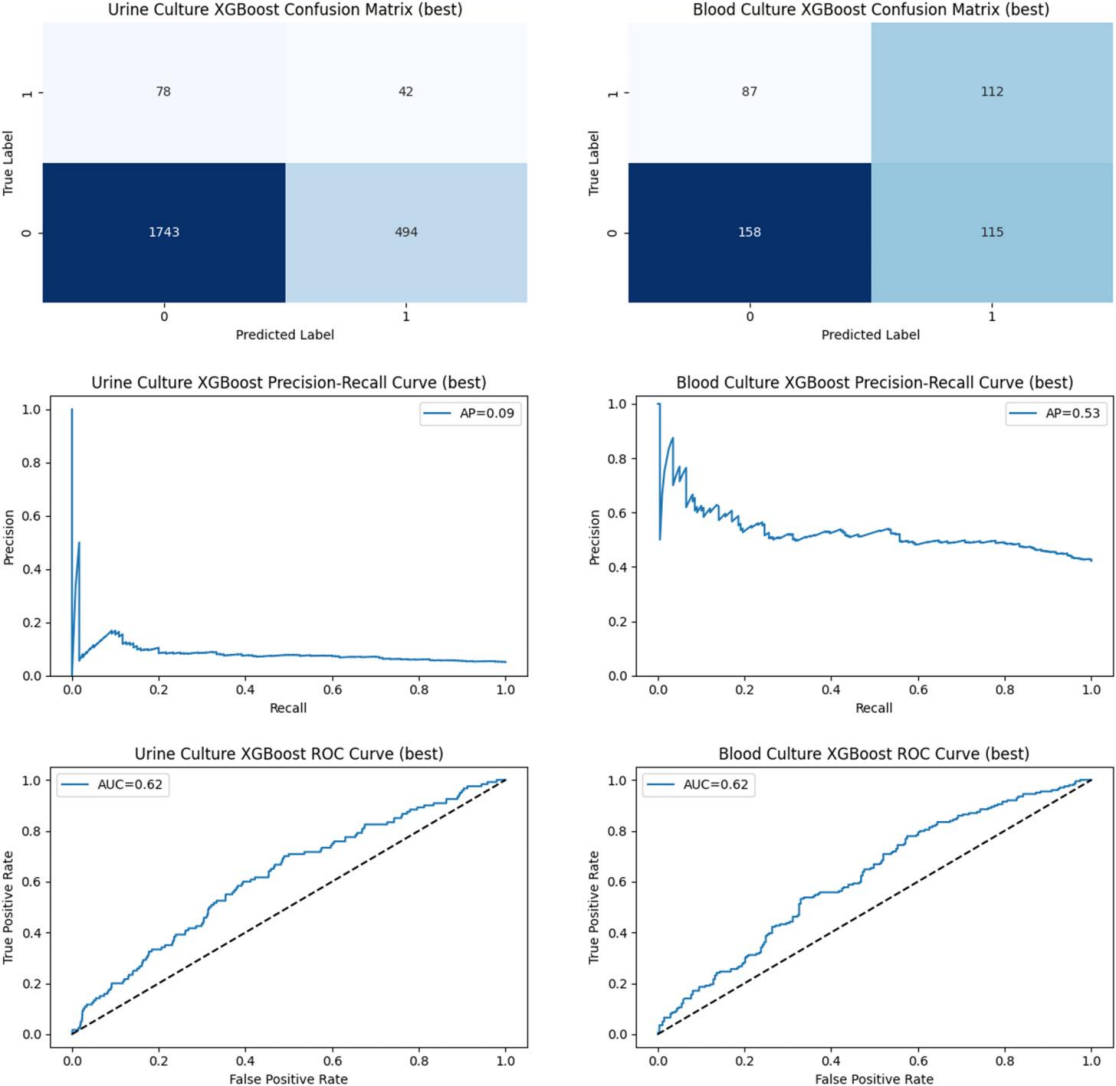
Confusion matrices (top), precision-recall curves (middle) and receiver operating characteristic curves (bottom) when the urine and blood culture models with the highest area under the receiver operating characteristic curve (AUC) values in the cross-validations (‘best’ models) were validated on the separate holdout urine (left) and blood (right) culture datasets. AUC values and average precision (AP) are included in inset boxes. Dotted lines in ROC plots represent an AUC of 0.5 (random predictions).

Figure 2 shows the distribution of ROC and precision-recall curves across the model cross-validations for the urine culture (cUTI versus uUTI) and blood culture (UTI versus non-UTI) training datasets, respectively. Table 2 shows the predictive performance when the trained models with the best cross-validation AUROCs were validated on the urine and blood culture holdout datasets. Figure 3 presents the ROC curves, precision-recall curves, and confusion matrices for model performance in this single validation run. The AUROC on the holdout validation dataset was 0.62 for both cUTI prediction in bacteriuria and for UTI prediction in bacteraemia. At a decision threshold of 0.5, F1 scores were low for both the urine and blood culture models (0.53 and 0.13, respectively)—the particularly low score in the urine culture dataset may have been due to improper labelling of complicated UTI due to limitations in clinical coding.

**Table 2.** Model performance: urine culture models and blood culture models

Metric	Urine culture model	Blood culture Model
Mean cross-validation performance in the training dataset		
AUROC	0.62	0.63
AP	0.09	0.56
F1	0.13	0.55
Corr	0.08	0.20
P	0.07	0.55
R	0.39	0.56
Acc	0.76	0.60
Performance of best cross-validation model on the holdout dataset		
AUROC	0.62	0.62
AP	0.09	0.53
F1	0.13	0.53
Corr	0.09	0.21
P	0.08	0.49
R	0.35	0.56
Acc	0.76	0.57

Metrics are AUROC (area under receiver operating characteristic), AP (average precision), F1 score, Corr (correlation), P (precision), R (recall), and (Acc) accuracy. All classes are determined by a threshold of 0.5.

### Fairness analysis and feature importance

Table 3 shows the evaluations of the models’ predictive performance across different demographic sub-groups to identify disparities in the corresponding AUROCs. The UTI versus non-UTI model performed similarly across racial groups. A small performance difference was observed between males and females. For age groups, it was observed that groups with fewer samples had lower AUROCs. The 30–39 age group had the highest AUROC overall. The fairness analysis revealed that while overall model performances were similar across demographic groups, some smaller groups had larger performance difference compared with the average value—this may be due to an insufficient sample size for robust model training.

**Table 3.** Fairness Metrics for the urine culture model and blood culture model

	Urine culture model			Blood culture model		
	AUROC	*n*	%	AUROC	*n*	%
White	0.62	6782	70.27	0.63	1262	68.33
Black	0.59	1303	13.5	0.61	290	15.7
Asian	0.73	249	2.58	0.63	58	3.14
Hispanic	0.60	398	4.12	0.67	89	4.82
Other	0.56	919	9.52	0.67	148	8.01
Male	N/A	0	0.0	0.63	841	45.53
Female	0.62	9651	100.0	0.63	1006	54.47
10–19	0.40	26	0.27	0.60	7	0.38
20–29	0.64	325	3.37	0.64	82	4.44
30–39	0.73	417	4.32	0.63	86	4.66
40–49	0.66	895	9.27	0.60	220	11.91
50–59	0.62	1368	14.17	0.58	358	19.38
60–69	0.57	1957	20.28	0.62	458	24.8
70–79	0.64	2124	22.01	0.57	382	20.68
80–89	0.61	1974	20.45	0.52	213	11.53
90–99	0.52	565	5.85	0.47	41	2.22

Based on number of subjects. Higher AUROC means the model has better performance for that demographic group.

**Figure 4.**
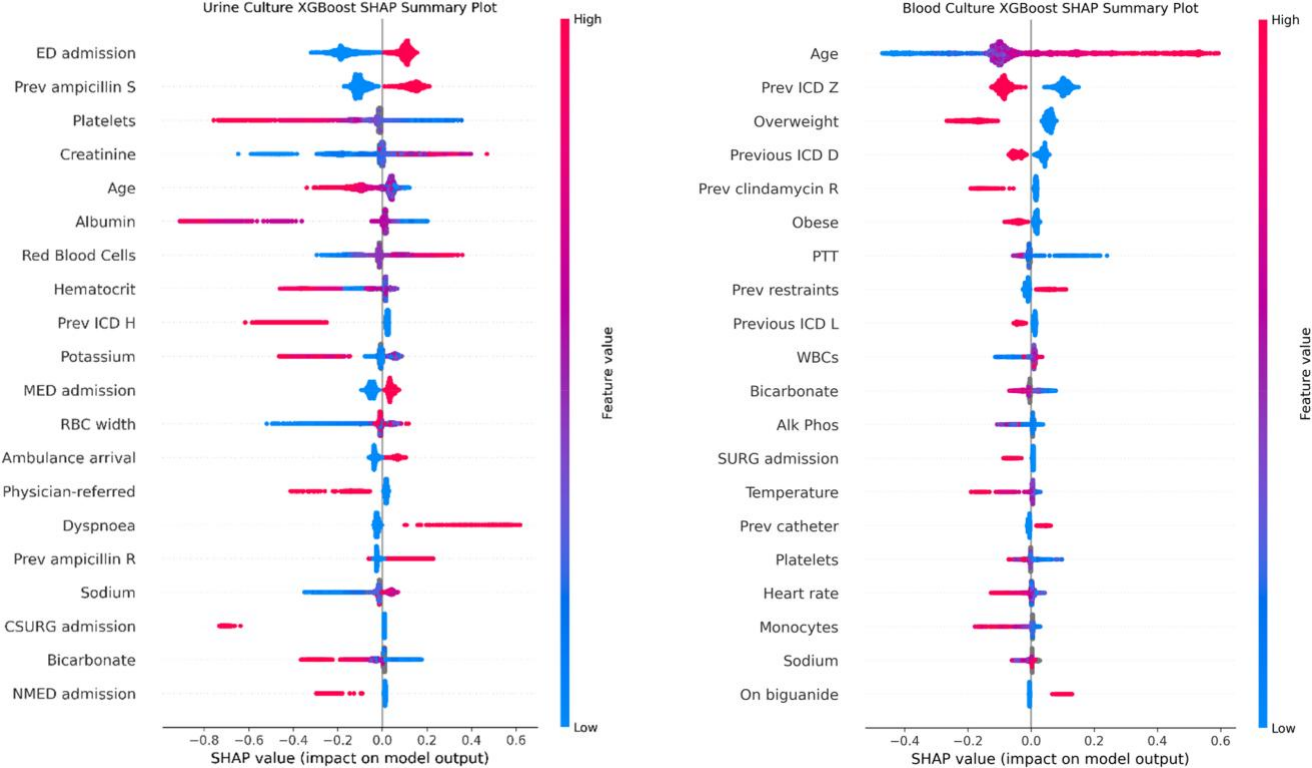
AP values for the 20 features with highest impact on model output in the best instances of the urine (left) and blood culture (right) XGBoost models in cross-validation. The Y-axis represents the features, ranked from the most important at the top to the least important at the bottom. The X-axis indicates the SHAP value, reflecting the contribution of each feature to the model’s prediction, where positive values (right of zero) increase the probability of the predicted outcome (e.g. complicated UTI or urinary focus of bacteraemia) and negative values (left of zero) decrease it. Each scatter point represents a data point coloured on a scale from blue (low feature value) to red (high feature value). For example, higher red blood cell counts (red points) increase the probability of complicated UTI, while lower counts (blue points) reduce the probability of complicated UTI in this dataset.

Figure 4 depicts SHAP values for the XGBoost models, which quantify the importance and direction (positive or negative) of each feature’s impact on the predictions made by the best model in the 10-fold cross-validation. Many of the features with higher importance were blood test results and observations. Age was a strong predictor of both cUTI and a urinary focus of bacteraemia. Admission via the emergency department was a strong predictor of cUTI, and healthcare exposure in general (International Classification of Diseases ‘Z’ Category, which corresponds to recording of previous diagnoses) was a strong predictor of a urinary focus of bacteraemia.

### Microsimulation study

**Figure 5.**
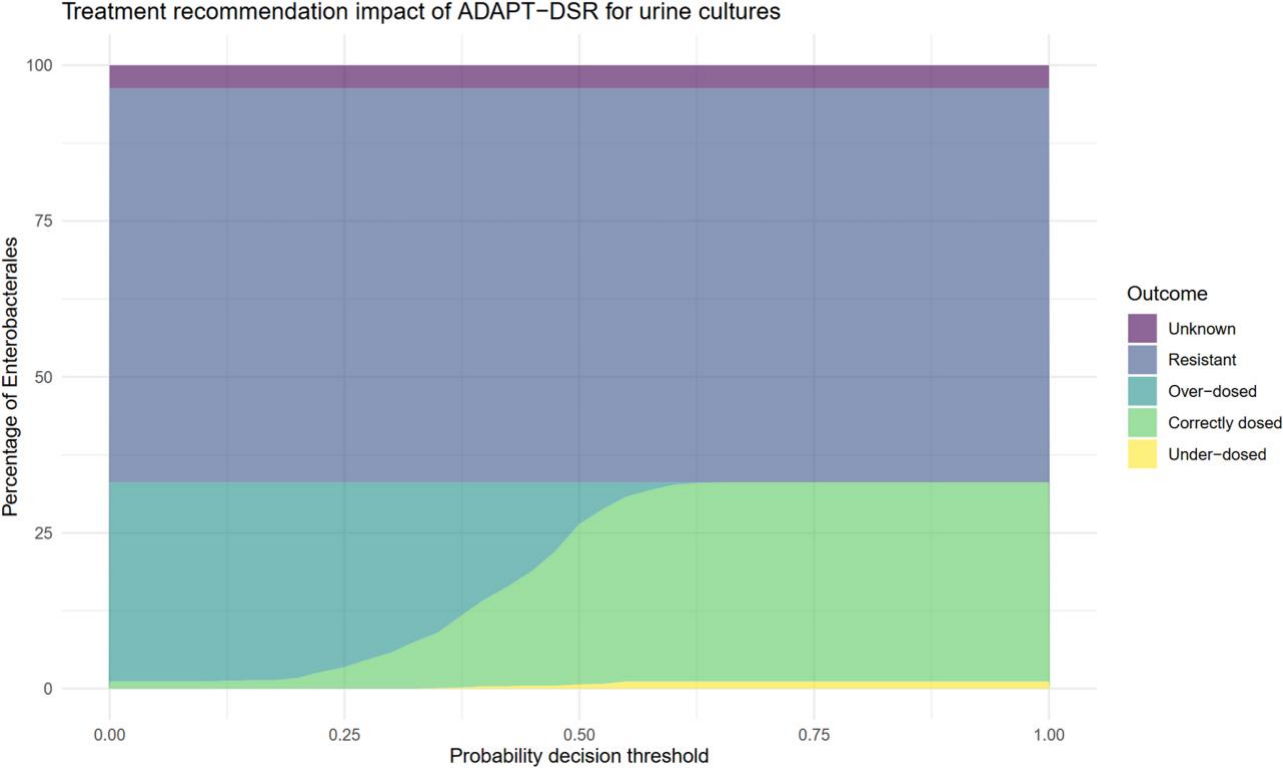
The appropriateness of urine culture amoxicillin dosing recommendations provided by the adaptive breakpoint susceptibility reporting algorithm (ADAPT-DSR) across a range of probability cutoffs (decision thresholds) for the model predicting complicated UTI. The percentages of different appropriateness outcomes in the microsimulation dataset are represented by shaded areas.

**Figure 6.**
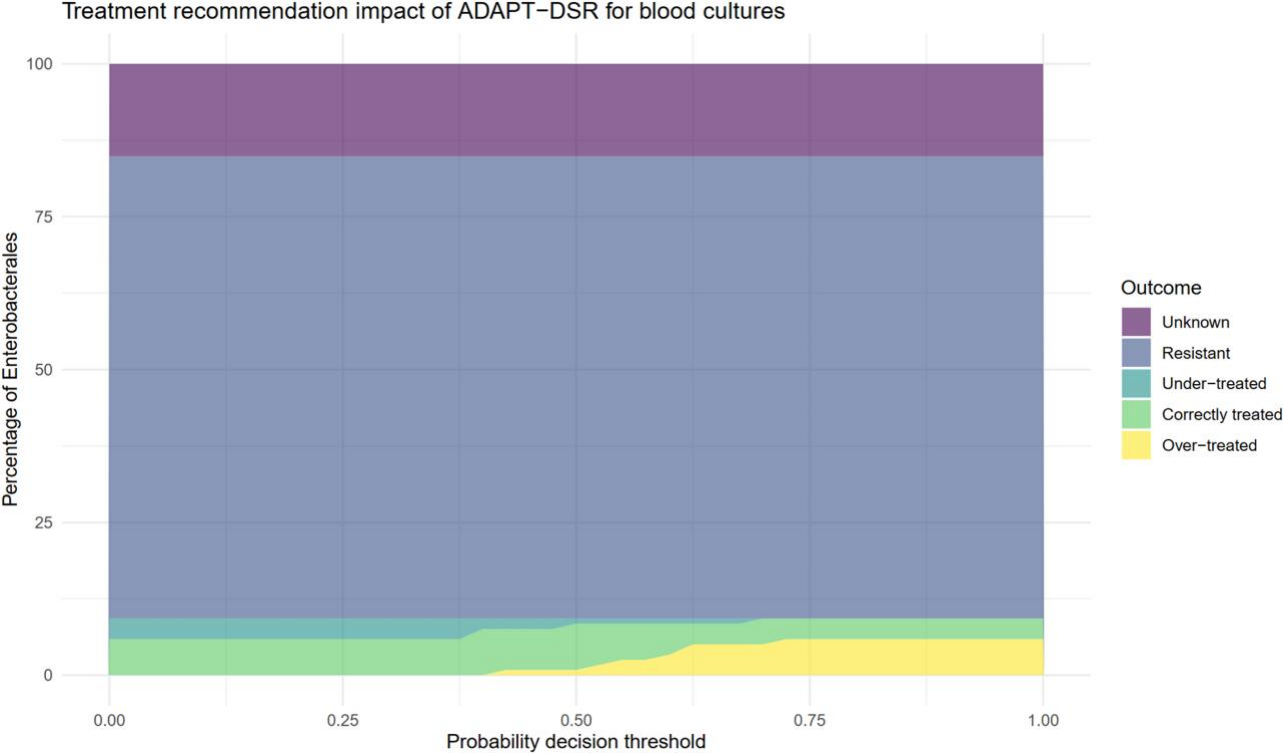
The appropriateness of blood culture amoxicillin treatment recommendations provided by the adaptive breakpoint susceptibility reporting algorithm (ADAPT-DSR) across a range of probability cutoffs (decision thresholds) for the model predicting UTI. The percentages of different appropriateness outcomes in the microsimulation dataset are represented by shaded areas.

The appropriateness of the algorithmically directed amoxicillin susceptibility reporting recommendations for Enterobacterales at a range of probability decision thresholds are displayed in Figures 5 and 6. For patients with Enterobacterales in their urine and at a decision threshold of 0.5, a total of 26% (*n* = 276) of patients were recommended the correct dose of amoxicillin according to EUCAST guidelines (79.3% of patients with ampicillin-susceptible isolates), 6.0% (*n* = 63) had a higher than necessary recommended dose of amoxicillin (because cUTI was predicted in a patient with uUTI), and 0.9% (*n* = 9) were recommended an insufficient dosage (because uUTI was predicted in a patient with cUTI). A total of 63.2% (*n* = 665) of Enterobacterales isolates were resistant to amoxicillin, and 3.7% (*n* = 39) had unknown amoxicillin susceptibility. Increasing the probability threshold at which a patient was classed as having cUTI to 0.73 (requiring a cUTI probability of at least 0.73 to predict cUTI, which was the decision threshold at which the proportion of correctly treated patients was the highest) further improved the proportion of correctly dosed patients (31.9%, *n* = 336%–96.6% of patients with ampicillin-susceptible isolates) at the expense of a small increase in the number of under-dosed patients.

For the blood culture model, at a decision threshold of 0.5, 6.7% (*n* = 8) of patients with Enterobacterales in their blood were recommended the correct dose of amoxicillin for their underlying source of infection (72.7% of patients with ampicillin-susceptible isolates), 1.6% (*n* = 2) were recommended a second agent unnecessarily (because a non-urinary focus of infection was predicted but the patient had a UTI), and 0.8% (*n* = 1) were inappropriately recommended amoxicillin monotherapy (because UTI was predicted but the patient had a non-urinary focus of infection). A total of 75.6% (*n* = 90) of isolates were resistant to amoxicillin, and 15.1% (*n* = 18) had unknown amoxicillin susceptibility. For bacteraemia, changing the probability threshold at which the patient was classed as having UTI did not increase the number of patients with the correct regimen recommended.

In the sub-group analysis of patients who were on aminopenicillin treatment in the 7 days following the culture specimen (25 patients with bacteriuria and 2 patients with bacteraemia), the approach recommended correct dosages in five patients prescribed amoxicillin–clavulanic acid and two patients prescribed amoxicillin. The approach incorrectly recommended high aminopenicillin doses in two patients prescribed amoxicillin–clavulanic acid and one patient prescribed amoxicillin. In two cases, patients were on amoxicillin and grew ampicillin-resistant Enterobacterales in their urine. Thirteen patients were on amoxicillin–clavulanic acid who grew ampicillin-resistant Enterobacterales in their urine, and two patients were on amoxicillin–clavulanic acid who grew ampicillin-resistant Enterobacterales in their blood—the appropriateness of recommendations for these cases could not be assessed because amoxicillin–clavulanic acid susceptibility testing was not performed. There were no patients on aminopenicillins who grew an ampicillin-susceptible organism in their blood.

In the additional analysis for nitrofurantoin breakpoint interpretation, the algorithm correctly recommended nitrofurantoin as a potential treatment based on patients’ predicted probability of complicated UTI in 35.0% (*n* = 826) of urine specimens. In 8.7% (*n* = 204) of cases, nitrofurantoin was incorrectly ruled out as a treatment option in uncomplicated UTI, and in 1.7% (*n* = 40) of cases, nitrofurantoin was incorrectly recommended as treatment in complicated UTI. In 33.3% (*n* = 784) of cases isolates were resistant to nitrofurantoin, and the remaining 21.3% (*n* = 503) of nitrofurantoin results were unknown.

## Discussion

This study provides proof-of-principle that EHR linkages and machine learning techniques could support individualized antimicrobial susceptibility reporting for blood and urine cultures, resulting in improved aminopenicillin dosing. The approach could enable the application of individualized breakpoints for a range of drug–pathogen–disease combinations, transforming the function of diagnostic microbiology laboratory information management systems into centres for data-driven healthcare interventions.

Our study suggests potential benefits in the use of machine learning approaches to apply individualized breakpoints; firstly, the approach facilitates the prediction of cUTI in patients without defining characteristics of cUTI (i.e. male sex, pregnancy etc.); secondly, machine learning techniques such as XGBoost provide feature importance information that can help tailor specific interventions—e.g. if blood test results and observations are important contributors to predictions, then blood tests and observations should be prioritized in these patient cohorts to optimize adaptive breakpoint reporting.

The implementation of our approach to support real-time patient care is somewhat challenging. Firstly, hardware and coding pipelines for secure EHR data linkage, cleaning, and storage are required—laboratory information management systems could provide a foundation for this, but considerable extra human resource is needed to maintain these systems and to extend them using health system-wide linked EHR data to reflect the movement of patients between providers.^21,22^ Secondly, our prediction models require further validation in other populations. Thirdly, site-specific decisions would need to be made regarding decision thresholds for algorithms, which depend on the risk appetites of different settings to potentially under- or over-treat patients based on the algorithm. Fourthly, the regulatory and governance requirements for application of the intervention need to allow machine learning algorithms to adapt to local settings. Lastly, for these interventions to be more widely used to facilitate breakpoint interpretation, the buy-in of key stakeholders such as EUCAST and the Clinical Laboratory Sciences Institute (CLSI) is essential. It may be that adaptive interventions such as this facilitate new approaches to breakpoint setting.

Our study has several limitations. Firstly, once the dataset had been partitioned for model training and non-relevant specimens/organisms/patients were excluded, relatively little data remained for the microsimulation dataset, particularly for the blood culture analysis (around 100 samples). Low rates of aminopenicillin susceptibility in Enterobacterales further limited numbers in the microsimulation study—the additional nitrofurantoin analysis, however, demonstrated the potential impact of adaptive breakpoint interpretation for an agent with higher susceptibility rates. We believe that paucity of data and class imbalance contributed to the relatively modest predictive value of the algorithms used—the study demonstrates that dataset sizes required for the assessment of targeted applications such as this one will be considerable. Further assessment of the approach in larger patient cohorts across multiple geographical areas will therefore be important to assess model performance—particularly for the urine culture model, where predictive accuracy for the more clinically severe minority class (complicated UTI) may need to be improved for patient safety reasons if decision threshold adjustment is insufficient. Similarly, we believe that disparities in model performance between different demographic groups is likely to have been driven by imbalances between the sizes of these groups—future work should therefore seek to access populations that are currently under-represented in this study to assess and minimize the potential risk of algorithmic bias. Secondly, clinical coding data were relied upon for outcome labels—such coding data may be incorrect, for example, underrepresenting complicated infections by labelling them only as UTI; thirdly, because coding data are only attached to admissions, we were only able to use information up to the first 24–48 h of admission to ensure we were not using subsequent data, following diagnosis—it is therefore likely that some potentially useful predictive information was missed, and the utility of the algorithm in predicting inpatient infection sources could be further optimized. A prospective study with manual review and time-accurate diagnosis coding by clinicians could facilitate future implementation by allowing true real-time adaptability, rather than only being able to adapt pathways to the admission level.

Despite these limitations, our study demonstrates proof-of-concept for how combining clinical prediction models with better data linkage between clinical microbiology laboratories and the bedside could help deliver personalized clinical breakpoint interpretation as a matter of routine, resulting in better patient outcomes through more appropriate antimicrobial use. To achieve its implementation, our approach requires further validation and investment in health data science infrastructure at regional, national, and international levels.

## Transparency declarations

Alex Howard declares personal consulting work for Pfizer outside the submitted work and a donation from Pfizer to the University of Liverpool for a public and professional engagement project outside the submitted work. William Hope holds or has recently held research grants with UKRI, EU (FP7, IMI-1, IMI-2), Wellcome, F2G, Spero Therapeutics, Antabio, Pfizer, Allecra, Bugworks, Phico Therapeutics, BioVersys, and Global Antimicrobial Research and Development Partnership (GARDP). He is (or has recently been) a consultant for Appili Therapeutics, F2G, Spero Therapeutics, Pfizer, GSK, Phico Therapeutics, Pulmocide, and Mundipharma Research Ltd. He was a member of the Specialist Advisory Committee for GARDP (2020–2023), a member of the British Society for Antimicrobial Chemotherapy (BSAC) Breakpoint Committee (2020–2023), a member of the Health Technology Appraisal (HTA) Prioritisation Committee for hospital care, and the Specialty National Co-lead for Infection for the National Institute of Health Research (NIHR) (2020–2024). Iain Buchan declares consulting fees via the University of Liverpool from AstraZeneca outside the submitted work. The remaining authors declare no competing interests.

## Code availability

The open-source code implementation is available on GitHub at https://github.com/AMRx-LIV/ADAPT-DSR.
